# A Convolutional Neural Network-Based Intelligent Medical System with Sensors for Assistive Diagnosis and Decision-Making in Non-Small Cell Lung Cancer

**DOI:** 10.3390/s21237996

**Published:** 2021-11-30

**Authors:** Xiangbing Zhan, Huiyun Long, Fangfang Gou, Xun Duan, Guangqian Kong, Jia Wu

**Affiliations:** 1 State Key Laboratory of Public Big Data, College of Computer Science and Technology, Guizhou University, Guiyang 550025, China; gs.xbzhan21@gzu.edu.cn (X.Z.); xduan@gzu.edu.cn (X.D.); gqkong@gzu.edu.cn (G.K.); 2 School of Computer Science and Engineering, Central South University, Changsha 410083, China; gff8221@csu.edu.cn; 3 Research Center for Artificial Intelligence, Monash University, Clayton, VIC 3800, Australia

**Keywords:** NSCLC, CNN, semantic features, transfer learning, dynamic sampling, sensors

## Abstract

In many regions of the world, early diagnosis of non-small cell lung cancer (NSCLC) is a major challenge due to the large population and lack of medical resources, which is difficult toeffectively address via limited physician manpower alone. Therefore, we developed a convolutional neural network (CNN)-based assisted diagnosis and decision-making intelligent medical system with sensors. This system analyzes NSCLC patients’ medical records using sensors to assist staging a diagnosis and provides recommended treatment plans to physicians. To address the problem of unbalanced case samples across pathological stages, we used transfer learning and dynamic sampling techniques to reconstruct and iteratively train the model to improve the accuracy of the prediction system. In this paper, all data for training and testing the system were obtained from the medical records of 2,789,675 patients with NSCLC, which were recorded in three hospitals in China over a five-year period. When the number of case samples reached 8000, the system achieved an accuracy rate of 0.84, which is already close to that of the doctors (accuracy: 0.86). The experimental results proved that the system can quickly and accurately analyze patient data and provide decision information support for physicians.

## 1. Introduction

Lung cancer is the second most common type of cancer in the world (11.4%) after female breast cancer (11.7%) and remains the leading cause of cancer-related deaths [1]. Based on the different histopathological characteristics of the tumor, lung cancer is classified into two main types: NSCLC and small cell lung cancer. The total number of patients with NSCLC accounts for approximately 85% of lung cancer cases [2]. Since patients with NSCLC have no obvious symptoms when the tumor is in its early stages, most NSCLC patients are already in the middle or late stages by the time they are diagnosed. The possibility of cure is very low, with an overall 5-year survival rate of only 15%. However, if patients are diagnosed early and receive effective treatment, the overall 5-year survival rate can be improved to 80% [3]. Therefore, the early-stage diagnosis of patients with NSCLC is very important in order to improve individual survival rate; however, to overcome this, we must develop a system which can aid doctors to diagnose, and make decisions, quickly and effectively.

In many developing countries in Asia, the health and lives of many patients are difficult to protect effectively due to underdeveloped medical technology, large populations, and the failure of patients to be diagnosed and receive effective treatment in a timely manner [4]. For example, China is a developing country, which faces the problem of an imbalance between its large geographical and population size when compared to the amount of available medical resources. In China, advanced medical equipment, technology, and resources are mainly located in large cities and more developed regions. These regions account for only 7% of the country’s land area and 22% of its population and retain 80% of its medical resources. However, the remaing 93% of the vast rural and underdeveloped areas, housing 78% of the population, receives only 20%, or less, of the available healthcare resources [5].

In many developing countries, in order to save patients’ lives without advanced medical resources, both doctors and patients have to face several dilemmas. These are as follows:

1. Heavy physician burden. The number of patients is large while the number of doctors is small. As a result, doctors are overwhelmed by the repetition of inefficient and cumbersome diagnostic tasks, making it difficult to attend to all patients and delaying the diagnosis or treatment of some patients.
2. Uneven geographical distribution of medical resources. While advanced medical resources are scarce and concentrated in large cities, most patients come from underdeveloped rural areas without better medical conditions or resources, which may delay their treatment, causing their condition to deteriorate.
3. Overtreatment due to doctor misdiagnosis. The lack of advanced medical testing technology and excellent qualified doctors can easily lead to misdiagnosis. Not only does this result in the burden of additional physical treatment as well as high and unnecessary medical costs for the patient, but it also worsens the doctor–patient relationship.

As the number of patients grows, the workload per physician continues to increase. When presented with a large number of case samples, physicians face the challenge of needing a lot of time and energy to handle the complicated diagnostic process and analyze the huge amount of case data as there is no fast and effective decision-making mechanism. To this end, artificial intelligence (AI)-based assisted medical systems can be built to address such problems. In recent years, with the development of 5G, mobile Internet, and AI-related technologies, many intelligent medical systems based on IoT, smart devices [6,7], big data and AI technologies [8], and medical sensors [9], developed to assist in diagnosis and decision-making, have emerged. AI techniques are now being used to some extent in radiation oncology [10] and cancer diagnosis [11]. These computer-aided diagnosis intelligent medical systems, based on advanced AI technologies [12], deep learning models [13], and integrated medical biosensors [14,15], have profoundly revolutionized the traditional medical technology industry, providing effective solutions to the aforementioned problems faced by physicians. In the medical Internet of Things [16], which combines the Internet of Things and sensors, the intelligent medical system can quickly detect and analyze the medical records of large amounts of lung cancer patients with powerful computing power, finding the hidden features in a variety of medical data [17], which provides doctors with accurate information support for disease diagnosis and treatment decisions [18]. This greatly reduces the workload of doctors and saves their time and energy, which in turn provides patients with convenient and fast medical services and reduces the rate of doctors’ misdiagnosis.

Currently, most of the hot research work in the field of smart medicine is to extract features from the medical images (CT, PET-CT) of patients through machine learning or deep learning techniques, then attempting to train the staging aid diagnosis model of lung cancer to provide clinical decision support for doctors. However, very few hospitals have large and expensive advanced medical equipment such as PET-CT, and the examination process is complicated and cumbersome. The electronic medical record in sensors for patients with NSCLC records a large amount of relevant diagnostic and decision pathology information, such as patient symptoms, signs, and relevant examination information. This data can be obtained through a variety of implantable, wearable, and invasive sensors on the patient’s body that provide continuous remote monitoring of life parameters. It is necessary to reasonably extract features from these medical record data and construct models to achieve more accurate disease diagnoses and to improve the efficiency of doctors’ diagnoses.

Semantic features of relevant pathological information can be automatically extracted from medical text data using natural language processing and deep learning algorithms [19] to train the assisted diagnosis models. Among them is CNN, which has the advantage of automatically extracting semantic features for text and then classifying it. Firstly, CNN can represent text semantically and extract features, which express words in the text as continuous dense vectors in multidimensional space, and words with similar semantics correspond to similar word vectors. Secondly, CNN is a locally connected network whose feature extraction is achieved by automatic learning. Therefore, convolution and pooling operations can be viewed as a local feature extraction process. When compared with traditional machine learning models, the cost of manually extracting features and the dependence of the effectiveness of the model’s implementation on the quality of manually extracted features are avoided.

However, many research works are missing important diagnostic and decision-making information when extracting pathological features from medical record data. In addition, the semantic feature extraction of text data is not accurate enough. More importantly, the model performance is affected by the unbalanced proportion of cases in the training dataset across the pathological stages of NSCLC, which results in less accurate diagnoses.

To address the above problems, this paper establishes an intelligent medical system for the assisted diagnosis and decision-making of NSCLC. The system obtains the patient’s medical record using sensors in real-time, converts it into corresponding text description data, and uses CNN deep learning technology to automatically extract semantic features and assist in the diagnosis of the patient’s cancer stage. To avoid missing important diagnostic and decision-making information, we pre-selected highly relevant tumor markers that accurately reflect the benignity and malignancy of lung cancer as the main training parameters—using multi-scale convolutional kernels to extract rich semantic features. Samples of patients with NSCLC cases were in the majority of those at more advanced stages. As a result, when training the early-stage prediction model, we used transfer learning techniques to migrate the parameters of the prediction models of most classes of large sample types to the prediction models of small sample types; therefore, achieving the transfer of shared knowledge between the samples of different stages of NSCLC, and avoiding the underfitting of the model due to an insufficient sample size. During the iterative training of the model, dynamic sampling techniques are used to construct positive and negative sample sets to form a balanced training data set, which in turn improved the prediction accuracy and robustness of the model.

The main contributions of this paper are:

1. The development of a new CNN-based assisted diagnosis and decision-making intelligent medical system with sensors, which can diagnose the staging of NSCLC patients and provide recommend treatment strategies to physicians by extracting semantic features from the text of highly relevant diagnostic and decision parameters. The system can be used to help physicians assess the effectiveness of patient treatment and adjust the next stage of the treatment plan in a timely manner according to the patient’s recovery.
2. The method of migrating the parameters of large-sample training models to small-sample training models using transfer learning techniques, which realizes the knowledge sharing and solves the impact on model performance caused by the problem of insufficient training samples.
3. The dynamic sampling technique training algorithm is proposed to construct a balanced training set of positive and negative samples for iterative training to improve the accuracy and robustness of the auxiliary diagnosis model.
4. The experimental data were all obtained from real-world NSCLC patient case samples recorded in three hospitals in China. The results show that our proposed new intelligent medical system can approach the diagnostic accuracy of NSCLC staging to the level of real doctors with good performance.

The rest of the article is prepared as follows. In Section 2, we discuss some related work. Section 3 describes the design of the system framework. Section 4 describes the experimental results and analysis. In Section 5, conclusions are presented, and future work is envisioned.

## 2. Related Work

Over the years, medical AI has become a hot topic in the research field, and many research publications based on advanced AI technologies have emerged discussing staging and subtype classification identification, early detection, survival analysis, and the assisted diagnosis of lung cancer.

The determination of the histopathological stage of lung tumors is of great importance to physicians in both the diagnosis and treatment of patients with NSCLC. In order to report a classification prediction of the pathological stage on CT images of patients with NSCLC, Yu et al. [20] used a random forest machine-learning algorithm, while Choi et al. [21] used U-Net and CNN to construct a cascaded neural network. In addition, Gou et al. [22] performed staging prediction on the text data of medical records of patients with NSCLC. These works can be further researched according to the stages of NSCLC, such as treatment strategy recommendations.

For the accurate classification identification of lung cancer and benign and malignant diseases, Yang et al. [23] showed high accuracy and feasibility using EfficientNet-B5 and ResNet-50 on histopathological whole slide images of multiple lung disease categories. On the other hand, Masud et al. [24] used modern deep learning CNN to build a classification and recognition framework for lung and colon cancer with an accuracy of 96.33%. Kriegsmann et al. [25] and Wang et al. [26] used multiple CNN models to experimentally evaluate histopathological images of NSCLC subtypes for classification. In particular, Han et al. [27], together with Chaunzwa et al. [28], verified that the CNN model VGG-16 outperformed other conventional machine learning algorithms in terms of the classification and recognition accuracy of NSCLC on PET/CT images. In contrast, Bębas et al. [29] identified NSCLC histological subtypes using PET/MRI texture analysis classification and achieved the best results (75.48%) among the many machine learning classifiers using support vector machines. However, these models only discriminate what type of lung cancer subtype is present, and there is room for expansion.

Regarding the screening and early risk prediction of whether a patient has lung cancer, Cheng et al. [30] proposed a new clinical decision support system for screening chest CT images for the presence or absence of lung nodules. Pandiangan et al. [31] used a feedforward neural network to train an artificial neural network model with patients’ physical symptoms as binary data labels. This detects the presence of lung cancer in the patient’s body with an accuracy of 96.67%. Xie et al. [32] used multiple metabolite data from patients, combined with multiple machine learning algorithms, to detect early lung cancer diagnostic biomarkers and found that five highly relevant metabolic biomarkers could be used for the early detection of lung cancer. Guo et al. [33] proposed a deep neural network framework to detect lung nodules from low-dose CT images and determine them benign or malignant to identify lung cancer with an accuracy of 99.02%. Accurately predicting the risk of malignant pulmonary lesions in pleural effusion allows for the early diagnosis of lung cancer [34]. Ahmad et al. [35] used a random forest, decision tree algorithm for the early diagnosis prediction of lung cancer by analyzing the data of multiple risk factors and pulmonary symptoms. The above studies illustrate the importance of AI technology for the early screening, risk prevention, and control of lung cancer in humans, focusing on preventive detection before the development cancer.

Due to the low 5-year survival rate of lung cancer patients, there is an urgent need for accurate survival analysis so that doctors can better diagnose and manage treatment for their patients. For this reason, survival analysis systems constructed with the Cox proportional hazards model (CPHM) as the backbone model have emerged [36,37,38]. In contrast, Huang et al. [39] used the XGBoost machine learning algorithm to build a model to predict the 1-year survival rate of NSCLC with bone metastases. Lu et al. [40] used U-Net segmentation to process hematoxylin-eosin (H and E) stained histological images of NSCLC and computer extracted tumor cell diversity features from them to predict the overall 5-year survival in early-stage NSCLC in combination with a CPHM. Lai et al. [41] developed a deep learning model combining gene biomarker expression and clinical data to predict the 5-year survival status of patients with NSCLC, showing high accuracy (AUC: 0.8163, accuracy: 75.44%). She et al. [42] used deep feedforward neural networks to integrate the CPHM for NSCLC to predict survival at 3 and 5 years and test the reliability of deep learning survival neural networks for individual treatment recommendations. However, survival analysis prediction is only a single aspect of lung cancer treatment management.

PET image preprocessing [43] has excellent recognition contrast for the tumor shape, size, and location of the cancer. It has been shown that radiomic features, based on PET/CT images, have a strong predictive power for NSCLC [44]. However, accurate postoperative prediction of NSCLC remains challenging. Lee et al. [45] developed a survival analysis model based on a multilayer perceptron with semi-supervised learning neural networks to predict the 3-year postoperative recurrence risk of NSCLC patients, which outperformed the CPHM. Ensemble learning has been applied to many relevant assisted medical systems. To shorten the pharmaceutical decision-making process, Luo et al. [46] used ensemble learning to construct a compound drug recommendation system for NSCLC that does not use gene sequence data. As a result, doctors were able to quickly select the appropriate drug for their patients with the help of the recommendation system. The ensemble learning-based assisted medical decision system for prostate cancer can help doctors in their diagnosis [47].

The above research work has shown that machine learning and deep learning have powerful predictive power for the classification and identification of diseases with high accuracy. However, each of the already proposed medical systems has room for expanded functionality. In comparison to the studies that have been presented, we developed an intelligent medical system with sensors, which is based on CNN to assist diagnosis and decision-making. This system can statistically analyze the medical record of NSCLC with sensors, not only to predict whether a patient’s disease is lung cancer or not but also to diagnose the stage of lung cancer patients and recommend treatment strategies to doctors.

## 3. System Design

### 3.1. Overall System Framework

Natural language processing techniques have found new applications in NSCLC assisted diagnosis and decision-making systems [48]. Intelligent medical systems, based on machine learning and deep learning technologies for assisted diagnosis and decision making, can help doctors accurately diagnose NSCLC patients, providing decision information for doctors to develop treatment strategies and evaluate the effects of treatment [49].

In order to provide effective diagnostic and decision-making information quickly, we pre-selected important parameters that are highly relevant indicators for lung cancer diagnosis, rather than directly processing massive amounts of raw data, to avoid losing important information. We also adopted multi-scale convolutional kernels to extract rich semantic features. These were used to transfer learning techniques and realize the transfer of shared knowledge from large samples to small samples, employing dynamic sampling methods to construct a balanced sample training set for the iterative training of the model.

**Figure 1:**
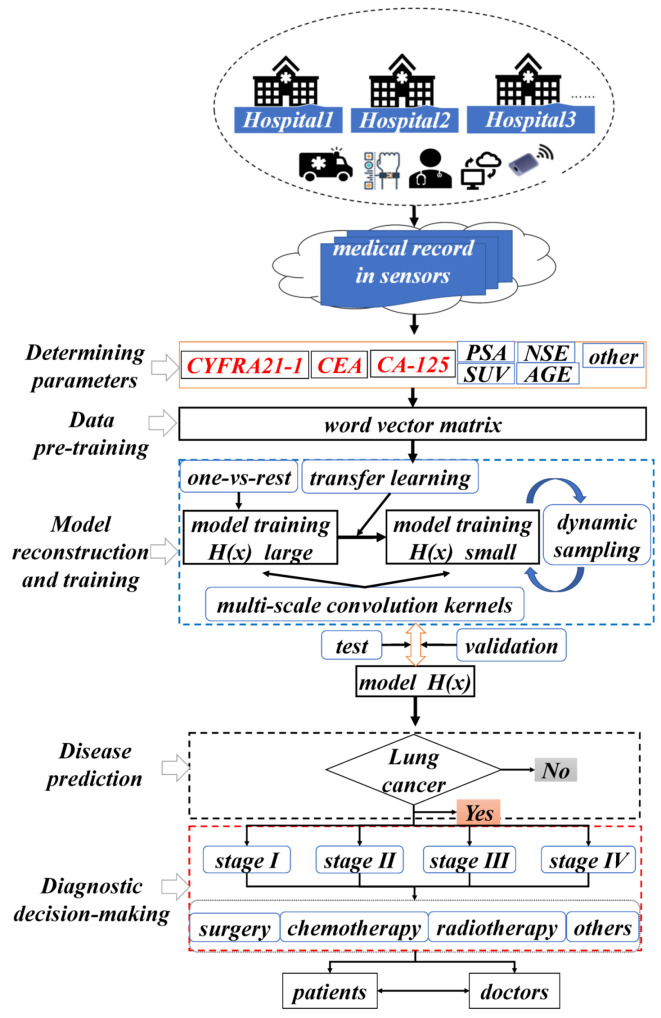
Framework diagram of the intelligent medical system used for the assisted diagnosis and decision-making of NSCLC.

During the diagnosis and decision-making process of NSCLC patients via intelligent medical systems, the general process consists of five parts (as shown in Figure 1): determining parameters, data pre-training, model reconstruction and training, disease prediction, and diagnostic decision-making.

Determining parameters: In the preliminary phase, when faced with a large sample of medical record data, physicians seeking to determine the pathological stage of tumors in patients with NSCLC may primarily focus on highly relevant diagnostic and decision parameters recorded in the medical record in sensors. Among these parameters, three tumor markers, erythropoietin 19 (CYFRA21-1), carcinoembryonic antigen (CEA), and carcinoembryonic antigen 125 (CA-125), play an important indicator role in determining the pathological stage of tumors and assessing the treatment effect in patients with NSCLC. To make the system prediction more accurate, other low-degree relevant indicator parameters recorded in the medical record in sensors, such as NSE, PSA, gender, age, symptoms, signs, years of smoking, and family history, are preprocessed and converted into training word vectors.

Data pre-training: In the second part, the medical record in sensors for NSCLC patients is pre-processed in a structured way. The textual word vectors are pre-trained using the Skip-gram model of word2vector to transform the selected diagnostic and decision parameters are into a low-dimensional data matrix that can be recognized by the system model.

Model reconstruction and training: In the third part, the system model is reconstructed and trained by dividing the dataset using One-Vs-Rest to address the sample class imbalance. This involves the use of transfer learning methods to deal with the problem of slow convergence of models due to the insufficient number of small samples. The processing of the sample training data takes a dynamic sampling approach to improve the robustness and generalization performance of the model.

Disease prediction: In the fourth part, we must first determine if the patient is an NSCLC patient. If so, we can proceed to the staging diagnosis. The trained system model will use CNN to extract and map semantic features to the word vector matrix of patient’s medical records, and use the four pathological stages (I, II, III, IV) of NSCLC, initial, early, intermediate, and terminal, as medical record sample category labels for the prediction.

Diagnostic decision-making: In the fifth part, the doctor will diagnose the disease based on the staging prediction made by the intelligent medical system on the text data of the new patient’s medical record,. The system will also recommend the corresponding treatment strategy to the doctor, as well as give the corresponding information back to the patient. In the case of stage I or II, the tumor of the NSCLC patient may be benign and highly operable, and surgery is the primary recommendation of the system. In the case of stage III or IV, the tumor is malignant, and the cancerous cells may have metastasized. In this case, the system recommends a comprehensive treatment strategy with radiochemotherapy as the main treatment, and surgery as adjuvant treatment.

After a phase of treatment for patients with NSCLC, the system can redo the stage-assisted diagnosis of three tumor marker text data, CYFRA21-1, CEA, and CA-125, which are highly relevant diagnostic and decision parameters recorded in the patient’s medical record in sensors. By comparing the staging decision values calculated by the system before and after treatment, doctors can determine whether a patient’s tumor has progressed from malignant to benign. If the staging decision value decreases significantly, it indicates that the treatment strategy recommended by the system is effective, and the next stage of the treatment plan can be adjusted. Otherwise, the patient’s condition should be further observed before deciding on a treatment strategy.

### 3.2. NSCLC Staging Prediction Model

Identifying the pathologic stage of NSCLC patients is an important prerequisite for doctors to develop effective treatment strategies, as the treatment strategies for different NSCLC pathologic stages are different. Firstly, we transformed the training set of four pathological stages of NSCLC into a binary classification training set of four pathological stages using the One-VS-Rest approach to train the prediction models for different NSCLC pathological stages. Then, the deep learning NSCLC staging prediction model was trained on the type with a high sample size, and the model parameters generated by the training are saved. Subsequently, when training the small-sample staging prediction model, the pathological stage with the highest number of co-occurrence with the small-sample pathological stage was selected. The knowledge of the model parameters of this majority class pathology stage was then transferred to the minority class pathology stage model training, which meant that the parameters of the majority class pathology stage prediction model were used as the initialization values of the minority class pathology stage prediction model. The dynamic sampling technique was also used to obtain a balanced data set to train the staging prediction model and improve the performance of the overall NSCLC staging prediction model.

**Figure 2:**
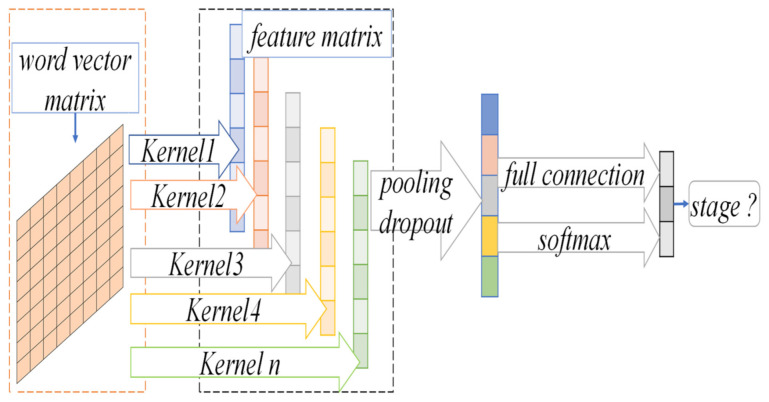
The model diagram of NSCLC staging prediction based on CNN.

In the CNN-based binary classification staging prediction model, the text in the medical record was split into word sequences by word processing. Then, word vectors of the words were pre-trained on medical-related diagnostic and decision parameter text data using Word2Vector’s Skip-gram model to represent discrete word symbols as semantic vectors in a low-dimensional continuous space. Next, after expressing each word of the case as its word vector, a two-dimensional word vector matrix expressing the case was obtained. Finally, the convolutional and pooling operations of CNN were used to extract features from the word vector matrix of cases and perform the binary classification of the four pathological stages. The structure diagram of the NSCLC staging prediction model based on CNN is given in Figure 2.

#### 3.2.1. The Skip-Gram Word Vector Model

The NSCLC medical record in sensors records natural language descriptions of the patients’ symptoms, signs, and histopathology-related examinations, such as serum, tumor markers CYFRA21-1, CEA, CA-125, etc., which contain important information for physicians to diagnose and make decisions about NSCLC patients. The text data of this important information is transformed into a data matrix by the Skip-gram model pre-trained word vector, which retains a large number of rich pathological semantic features. Then, the CNN deep learning model extracts key pathological semantic features to predict the pathological stage type of NSCLC patients.

The Skip-gram model is a model that takes the current word $W_{c}$ as the central target word and generates contextual words in its front and back d window after a projection layer. In the preprocessing phase of the case text data, CYFRA21-1 is selected as $W_{c}$ and the context window d is set to projectively generate the natural language description words around it. Therefore, the closer the distance, the higher the conditional probability of generation, and the vector representation of CYFRA21-1 is obtained by maximizing the log-likelihood function to train the model, as well as other diagnostic and decision parameters.

(1) $$LL=\underset{\gamma \in G\left(W_{c}\right)}{\sum }\log p(\gamma |W_{c})$$

**Figure 3:**
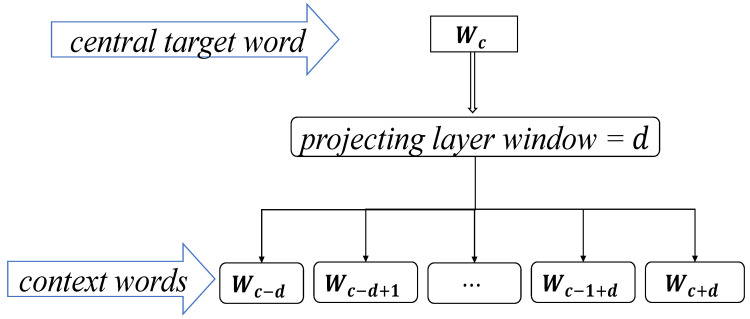
The structure diagram of Skip-gram model.

Equation (1) shows the maximized log-likelihood function. where p represents the conditional probability of generating a context word γ with the given target center word $W_{c}$, and $G\left(W_{c}\right)=\left\{W_{c-d},W_{c-d+1},\cdots ,W_{c-1+d},W_{c+d}\right\}$ denotes the set of all context words γ within window d. The structure diagram of the Skip-gram model is shown in Figure 3.

#### 3.2.2. CNN Convolution Operation

The CNN-based prediction model for NSCLC staging proposed in this paper is shown in Figure 2. The model contains a convolutional layer and a pooling layer. Firstly, a convolution operation is performed on the two-dimensional feature matrix, where the length of the convolution kernels coincides with the length of the word vector and each convolution kernel produces a column vector representation. Secondly, a maximum pooling method is used for each column vector to select the maximum value as the output. Thirdly, the maximum values of all column vectors are formed into a vector of fixed dimensions in order, and the length of the vector is the same as the number of convolution kernels, called the feature vector. Finally, the pooled nodes are classified as fully connected.

Suppose an n dimensional vector is used to represent the word vector, and $v_{i}\in {\mathbb{R}}^{n}$ denotes the word vector representation of the i-th word. The patient medical record contains num words, and the patient medical record can be represented as $V_{1:num}$, where ⊕ denotes the vector join operation, as shown in Equation (2).

(2) $$V_{1:num}=v_{1}⊕v_{2}⊕\cdots ⊕v_{num}$$

After generating a two-dimensional matrix representation of the text, the result is input to the convolutional layer, and the convolutional kernel is used to extract semantic features from the training data. Given (1) $V_{i:i+m-1}$ denotes the window vector-matrix from the i-th word to the i+m−1-th word in the word sequence. (2) A convolution kernel matrix $k\in {\mathbb{R}}^{hn}$ with the aim of acting the convolution kernel k on m successive word vectors is used to produce an output result.

**Figure 4:**
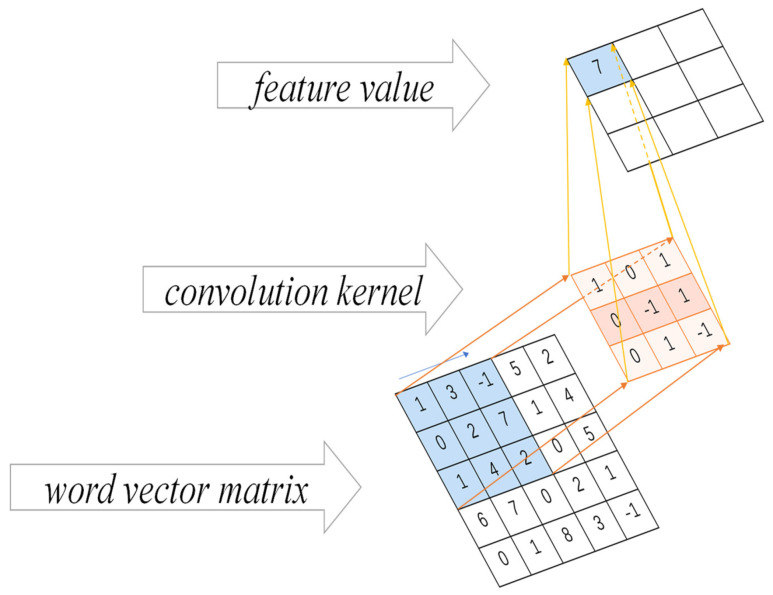
The diagram of convolution operation.

The convolution operation is shown in Figure 4. The result $a_{i}$ produced by the convolution kernel k acting on $V_{i:i+m-1}$*,* can be calculated as in Equation (3).

(3) $$a_{i}=f\left(k\cdot V_{i:i+m-1}+b\right)$$

where f(∗) is usually a nonlinear function, which can be a ReLU function, *tanh* function, etc., k is the above convolution kernel, and b is the bias term.

To extract a richer data representation of diagnostic and decision parameters in the case text, the model uses convolutional kernels with multiple windows to obtain more semantic information. After the convolution layer, feature maps with dimensions of varying sentence length are generated. These are usually of large dimensions and difficult to train a suitable classification model directly. These feature maps are used to input the pooling layer for dimension reduction, and at the same time, capture the most important information. In this paper, the model uses the maximum pooling method, and, as a result, the maximum pooling outputs the maximum value in the feature map. After the pooling layer, a fixed-length feature vector is generated (the length is the same as the number of convolutional kernels), and the feature vector is input into the fully-connected classification layer for sample classification.

### 3.3. Prediction Model for NSCLC Staging Based on Transfer Learning and Dynamic Sampling for Small Samples

When there is an imbalance in the training data across pathological stage categories, the CNN tends to classify the samples into the label categories with larger sample sizes in the training data. In the binary classification training data for each pathological stage, the positive sample set is the case sample of that pathological stage, and the negative sample set is the case sample of all other pathological stages. The number of negative samples is much larger than the number of positive samples, resulting in a low recall rate when predicting the stage of NSCLC.

In addition, the sample of cases at each pathological stage were uneven, with a larger number of cases at stage III or IV and a smaller number of cases at stage I or II. Moreover, the degree of imbalance in the amount of training data varies widely across pathological stage categories. The imbalance is particularly severe in the small-sample pathological period case data, which are much less likely to be selected into the training set than the large-sample pathological stage data. Therefore, the model’s underfitting learning of small sample case data leads to the low recall of a few classes of pathological stage prediction models, which affects the performance of multi-pathological stage label prediction for NSCLC and cannot meet clinical use.

**Figure 5:**
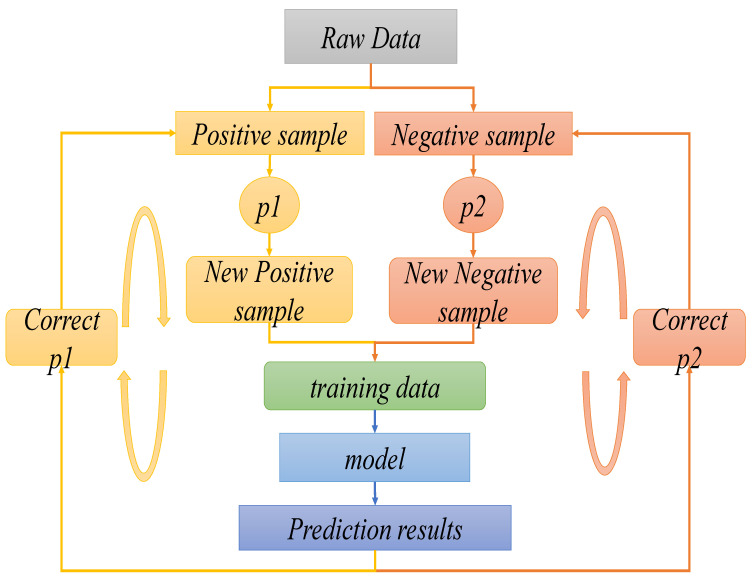
The prediction model diagram of NSCLC based on dynamic sampling technique for small samples.

First, the efficient staging prediction model was trained on a large sample of pathological stage cases with a high co-occurrence frequency with the present pathological stage and a sufficient data set. The large sample NSCLC staging prediction model was used as the initialization value for the small sample model. Then, the NSCLC staging prediction model was retrained on the small sample data set. The set of training samples is selected by dynamic sampling during training, the positive and negative samples are sampled separately. Finally, the sampled datasets are merged and used as the training data set for the next round, as shown in Figure 5. After each iteration of training, the sample sampling probability is updated according to the model’s prediction results on the sample set, the sampling probability of samples with classification errors and those with low confidence in classification is increased, thus a balanced training data set is constructed by dynamic sampling to train the model.

The training steps of the proposed algorithm (Algorithm 1) for predicting the pathological stage of NSCLC with small samples, combining transfer learning and dynamic sampling, are shown below:

**Algorithm 1:** The dynamic sampling technique training algorithm **Input:**
$S=\left\{\left(x_{1},y_{1}\right),\left(x_{2},y_{2}\right),\cdots ,\left(x_{n},y_{n}\right)\right\},x_{i}\in V\subseteq {\mathbb{R}}^{n},y_{i}\in \left\{l_{1},l_{2},\cdots ,l_{n}\right\}$ is the multi-label data set. where n is the total number of labels, NSCLC has four stages, so n=4, the labels to be trained are $l_{i}\in \left\{I,\mathrm{II},\mathrm{III},\mathrm{IV}\right\}$, the number of iterations is E and the size of the training data block for each iteration is M. **Output:** The prediction model $H_{i}\left(x\right)$. **Step 1:** For any label $l_{j}$, the co-occurrence frequency $F\left(l_{i},l_{j}\right)$ of the small sample pathological stage label $l_{i}$ and $l_{j}$ is calculated, as shown in Equation (4). Then, the parameters of the training model for the large sample case dataset are selected and saved according to the label $l_{j}^{max}$ corresponding to the maximum $F\left(l_{i},l_{j}\right)$ value, which is used as the initialization value for the small sample pathological period prediction model. where $Q\left(\left\{l_{i},l_{j}\right\}\subseteq y_{n}\right)$ is a binary function that labels each case sample of NSCLC patients, and the labeling value is 1 if $l_{i}$ and $l_{j}$ have appeared in the same case sample, otherwise the labeling value is 0, as shown in Equation (5).
(4)$$F\left(l_{i},l_{j}\right)=\underset{\left(x_{n},y_{n}\right)\in S}{\sum }Q\left(\left\{l_{i},l_{j}\right\}\subseteq y_{n}\right)$$
(5)$$Q\left(\left\{l_{i},l_{j}\right\}\subseteq y_{n}\right)=\{\begin{array}{c} 1,\;\;\;\;l_{i}\in y_{n}\;and\;l_{j}\in y_{n}\\ 0,\;\;\;\;l_{i}\notin y_{n}\;or\;l_{j}\notin y_{n} \end{array}$$
 **Step 2:**
S is split into multiple binary data sets $\left\{S_{1},S_{2},\cdots ,S_{k}\right\}$ of NSCLC pathological stages using the One-VS-Rest approach, where $S_{i}$ is the training set of the pathological stage label $l_{i}$. Then, select the majority pathological stage label $l_{k}$ with the most frequent co-occurrence with the pathological stage label $l_{i}$. Train the large sample NSCLC pathological stage prediction model $H_{k}\left(x\right)$ using the training dataset $S_{k}$ with label $l_{k}$ (see Equation (6)), and save the parameters of $H_{k}\left(x\right)$.
(6)$$H_{k}\left(x\right)=T\left(S_{k}\right)$$
 **Step 3:** The parameters of the pathological stage prediction model $H_{k}\left(x\right)$ for NSCLC in a large sample of cases are read as the initialized model $H_{i,1}\left(x\right)$ for the pathological stage label $l_{i}$. The majority category sample set of $l_{i}$ is $S_{k,neg}$, the minority category sample set is $S_{k,pos}$, the sample size is $N_{neg}$ and $N_{pos}$, respectively, with a total sample size of N. Initialize the minority category sample sampling probability $P_{i,1}=\left\{P_{i,1}\left(1\right),P_{i,1}\left(2\right),\cdots ,P_{i,1}\left(N\right)\right\}$, as shown in Equation (7). Since the sum of the sampling probabilities of both positive and negative samples is M/2, after sampling for each positive and negative sample according to the sampling method in step 4, the average value of the number of positive and negative samples can be obtained by sampling M/2, so the samples constructed by sampling are balanced.
(7)$$P_{i,1}\left(j\right)=\{\begin{array}{c} \frac{M}{2\times N_{pos}},\;\;\;\;l_{i}\in y_{j}\\ \frac{M}{2\times N_{neg}},\;\;\;\;l_{i}\notin y_{j} \end{array}$$
 **Step 4:** The positive and negative sample sets are sampled separately based on the sampling probability $P_{i,t}$. For any sample $\left(x_{j},y_{j}\right)$ with a sampling probability of $P_{i,t}\left(j\right)$, a random value $R\left(x_{j}\right)$ with a uniform distribution between 0 and 1 is generated using R(∗). If $R\left(x_{j}\right)\leq P_{i,t}\left(j\right)$, the sample $\left(x_{j},y_{j}\right)$ is added to the new balanced sample set $S_{i,train}$. At this point, if $l_{i}\notin y_{j}$, the sample $\left(x_{j},y_{j}\right)$ will be added to the partial majority class sample set $S_{i,neg}^{sel}$. On the contrary, the sample $\left(x_{j},y_{j}\right)$ will be added to the minority class sample set $S_{i,pos}$ if $l_{i}\in y_{j}$, as shown in Equations (8) and (9). For each sample $\left(x_{j},y_{j}\right)$, its sampling probability is $P_{i,t}\left(j\right)$, which is equal to the probability that the randomly generated number $R\left(x_{j}\right)$ is smaller than $P_{i,t}\left(j\right)$. Therefore, when $R\left(x_{j}\right)$ is smaller than $P_{i,t}\left(j\right)$, the sample $\left(x_{j},y_{j}\right)$ is added to this balanced sample set, and it is reasonable to update the sampling probability using this algorithm.
(8)$$S_{i,neg}^{sel}=\{\left(x_{j},y_{j}\right)|R\left(x_{j}\right)\leq P_{i,t}\left(j\right),\left(x_{j},y_{j}\right)\in S_{i,neg}\}$$
(9)$$S_{i,pos}^{sel}=\{\left(x_{j},y_{j}\right)|R\left(x_{j}\right)\leq P_{i,t}\left(j\right),\left(x_{j},y_{j}\right)\in S_{j,pos}\}$$
 Finally, $S_{i,neg}^{sel}$ and $S_{i,pos}^{sel}$ are combined to form the training set $S_{i,train}$:
(10)$$S_{i,train}=S_{i,neg}^{sel}\cup S_{i,pos}^{sel}$$
 **Step 5:**
$H_{i,t-1}\left(x\right)$ is trained based on the set data $S_{i,train}$ to generate the new model $H_{i,t}\left(x\right)$, as shown in Equation (11).
(11)$$H_{i,t}\left(x\right)=T\left(H_{i,t-1}\left(x\right);S_{i,train}\right)$$
 **Step 6:** If result of calculating the probability that the predicted sample of model $H_{i,t}\left(x\right)$ on the overall training sample of a positive sample is $\eta_{i,t}$ and $\eta_{i,t}\left(j\right)\in \left[0,1\right]$, this indicates that the probability value the predicted sample of the classifier belongs to a positive sample. The larger $\eta_{i,t}\left(j\right)$ is better for positive samples, and the smaller $\eta_{i,t}\left(j\right)$ is better for negative samples. $\eta_{i,t}$ can be used to update the sampling probability $P_{i,t+1}=\left\{P_{i,t+1}\left(1\right),P_{i,t+1}\left(2\right),\cdots ,P_{i,t+1}\left(N\right)\right\}$, as shown in Equation (12).
(12)$$P_{i,t+1}\left(j\right)=\{\begin{array}{c} P_{i,t}\left(j\right)exp(1-\eta_{i,t}\left(j\right)),\;\;\;\;l_{i}\in y_{j}\\ P_{i,t}\left(j\right)exp(\eta_{i,t}\left(j\right)),\;\;\;\;l_{i}\notin y_{j} \end{array}$$
 When the model $H_{i,t}\left(x\right)$ predicts the training sample incorrectly, or correctly but with low confidence, sampling probability of that sample is increased, which increases the focus of the model on the sample. Conversely, when the model predicts a sample correctly and with high confidence, it relatively reduces the sampling probability of the sample which reduces the attention of the model to it. This will increase the distinguishability of the model for positive and negative samples to improve the prediction accuracy and confidence of the model. Therefore, when the sample $\left(x_{j},y_{j}\right)$ is a positive sample, the closer $\eta_{i,t}\left(j\right)$ is to 0, the probability of the updated sample increases when the classification is incorrect or correct but the confidence is low. When it is a negative sample, the closer $\eta_{i,t}\left(j\right)$ is to 1, the probability of updated sampling will increase when the classification is incorrect or correct but the confidence level is not high. The sampling probabilities of positive samples are regulized, where $Sum_{t,pos}$ is the sum of all positive sample sampling probabilities, as shown in Equations (13) and (14).
(13)$$P_{i,t+1}\left(j\right)=\frac{M\times P_{i,t+1}\left(j\right)}{2\times Sum_{t,pos}}$$
(14)$$Sum_{t,pos}=\underset{\left(x_{n},y_{n}\right)\in S_{i,pos}}{\sum }P_{i,t+1}\left(n\right)$$
 Similarly, the sampling probability of negative samples are regularized, where $Sum_{t,neg}$ is the sum of the sampling probabilities of all negative samples, as shown in Equations (15) and (16), respectively.
(15)$$P_{i,t+1}\left(j\right)=\frac{M\times P_{i,t+1}\left(j\right)}{2\times Sum_{t,neg}}$$
(16)$$Sum_{t,neg}=\underset{\left(x_{n},y_{n}\right)\in S_{i,neg}}{\sum }P_{i,t+1}\left(n\right)$$
 **Step 7:** Determine whether the specified number of iterations is reached and return the final classifier if it is satisfied; otherwise, continue with steps 4 to 7 using the new sampling probabilities.

So far, after continuous iterative model reconstruction training, the prediction of the model H(x) is more accurate, and the performance is more stable. The trained model is used to construct an intelligent medical system based on CNN for the assisted diagnosis and decision-making of NSCLC. For the undiagnosed case sample, the physician evaluates the authenticity of the patient’s diagnosis and the feasibility of the treatment strategy based on the decision value of the pathological stage of the NSCLC patient calculated by the system, which is the prediction confidence of the model H(x), the predicted stage, and the recommended treatment strategy, combined with their own experience. After implementing the treatment, the system re-performs the above process on the patient’s symptoms, signs, tumor marker tests, and other text data from the medical record in sensors. The doctor determines the patient’s physical recovery accordingly and adjusts the next treatment plan. After each phase of treatment, doctors use the assistive intelligence medical system to calculate and analyze, track the patient’s condition, and adopt the appropriate treatment plan to avoid misdiagnosis or over-treatment, thus saving time and saving the patient’s life.

## 4. Experiments and Analysis

### 4.1. Diagnostic Data Parameters

All experimental case data in this paper were collected from three hospitals in China from 2011 to 2015. All patients were aged 45–60 years old, and the data statistics over a five-year period are shown in Table 1.

During the diagnosis of NSCLC, physicians focus mainly on important, relevant reference indicators in order to be able to make efficient decisions. Therefore, the three highly relevant diagnostic and decision parameters with the highest weights in our proposed assisted intelligent medical system are selected: CYFRA21-1, CEA, and CA-125. The values of these three tumor marker parameters are important for disease detection, pathological stage determination, evaluation of treatment efficacy, and the prognosis of patients with NSCLC. Table 2 show the normal ranges of these three diagnostic and decision parameters.

In order to extract more comprehensive semantic features of the medical record text in the sensor, and thus improve the accuracy of the model, other low-degree relevant diagnostic parameter indicators are considered, such as other relevant examination indicators, patient symptoms, etc. Among them, the normal ranges of five low-degree relevant diagnostic and decision parameters are shown in Table 3.

After model training, the system model H(x) calculates the decision value for the pathological stage of the case when sensors transmit the text data information of the detected new NSCLC patient cases to the assistive diagnosis and decision-making intelligent medical system. Based on a statistical analysis of data trained on a large number of case samples in the pre-model period, a range of decision values for the classification of four pathological stages of NSCLC was thus determined, as shown in Table 4. 

If the decision value calculated by the system is less than 18, the patient is in good health and NSCLC is ruled out, but further tests are needed and confirmed. If the decision value is greater than 18 and less than 57, the patient’s NSCLC tumor is now at Stage I. Again, for decision values within 58–119, the patient is in stage II of NSCLC. Correspondingly, if the decision value is in the Stage III range, the patient is more severely ill at that point. If the decision value is greater than 180, the patient’s NSCLC has progressed to Stage IV, indicating that the patient’s disease has deteriorated to a very serious level.

Table 5 show the mean data recorded for each examination parameter for the sample of three groups of similarly symptomatic cases selected at random from undiagnosed patients. Each group includes several patients, five examinations are performed for each group separately. After each examination, the data of each parameter for each patient in the group are examined. Then, the average value of the corresponding parameters of the group is calculated. These parameters include six other check parameters in addition to three highly relevant diagnostic and decision parameters.

**Figure 6:**
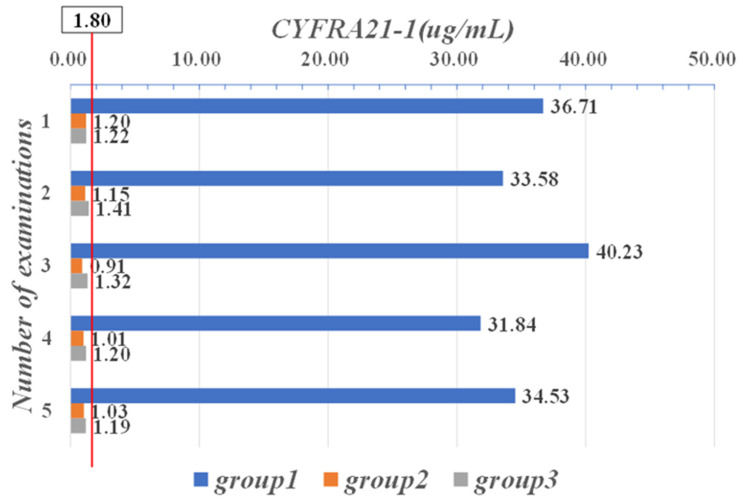
Mean data of five CYFRA21-1 examinations in three groups of patients.

Figure 6 show a comparison of the mean data between these three groups, for the five CYFRA21-1 examinations performed on the patients. In the second and third groups, the mean CYFRA21-1 values were within the normal range [0, 1.80], whereas the patients in the first group had mean CYFRA21-1 values of more than 30 μg/mL in all five examinations, indicating that the patients in this group had more severe illness.

**Figure 7:**
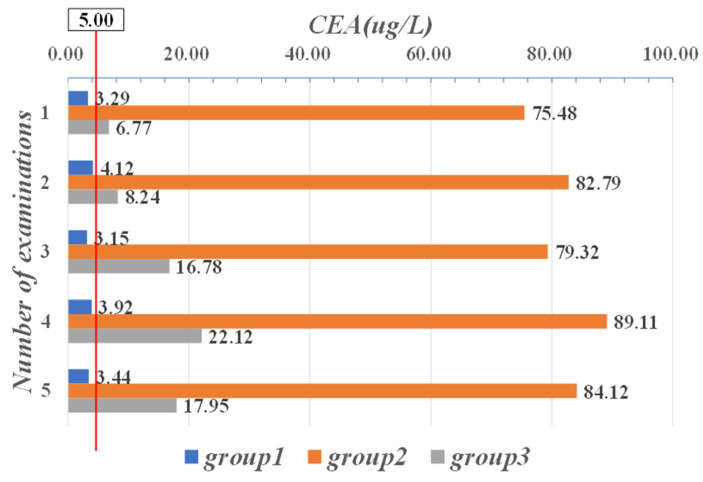
Mean data of five CEA examinations in three groups of patients.

As shown in Figure 7, the mean CEA data for the first group of patients were normal for all five examinations. In contrast, in the second and third groups, the mean of five CEA examinations was outside its normal range in both groups [0, 5.00]. In particular, the values in the second group all exceeded the maximum critical value (50 μg/L) of the normal range by more than ten times, which means that the patients in this group were seriously ill.

**Figure 8:**
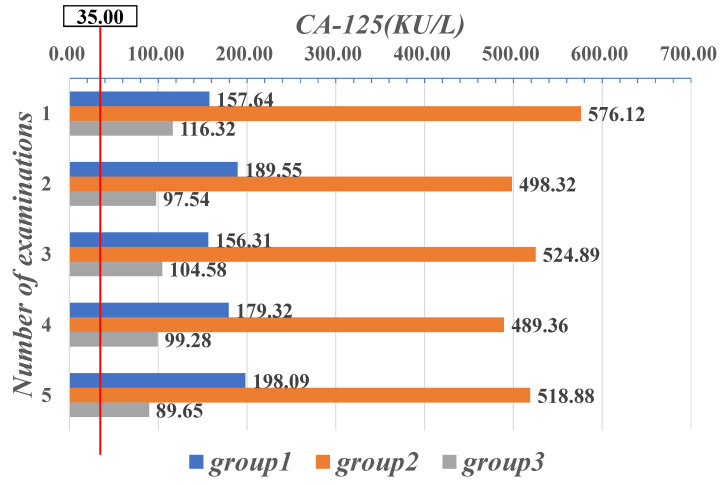
Mean value data of five CA-125 examinations in three groups of patients.

In all three groups, the mean values of the patients’ CA-125 examined were over the normal range [0, 35.00]. In particular, the mean CA-125 values of the patients in the second group were all over 480 (KU/L), which means that the mean values were more than ten times greater than the highest threshold of their normal range, as shown in Figure 8. It is clear that patients in the second group had mean values for both diagnostic and decision-making parameters, CEA and CA-125, that were well outside their respective normal ranges, which indicates that the patients’ conditions may have deteriorated severely or even progressed to the advanced stage of the disease.

**Figure 9:**
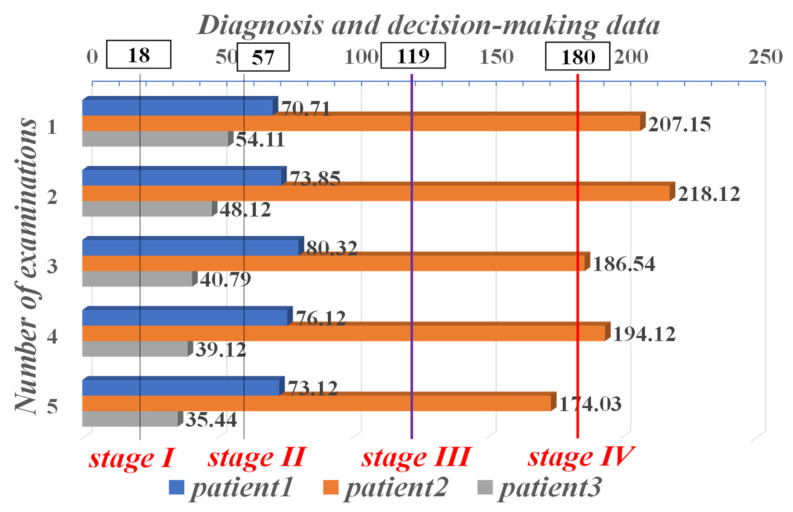
Auxiliary system staging diagnostic results for three representative patients in three groups with five examination data.

It is difficult to diagnose exactly what stage the NSCLC patient is at from a single index parameter. Therefore, we performed a joint computational analysis of three highly relevant diagnostic and decision-making parameters: CYFRA21-1, CEA, and CA-125. In each of the three groups, one representative patient was randomly selected, and text data from the five examination records of the three selected representative patients with highly relevant diagnostic and decision parameters were used to calculate decision values for staging diagnosis by the NSCLC assisted diagnosis and decision intelligent medical system. The results of the system diagnosis are shown in Figure 9. The representative patient selected from the first group, patient1, was in Stage II of NSCLC. In contrast, patient2, a representative patient in the second group, was very ill and was in Stage IV. The mean values of CEA and CA-125 in this group of patients were each more than ten times beyond the maximum threshold of its normal range. The representative patient in the third group, patient3, was in Stage I of NSCLC, indicating that the patients in this group had milder conditions.

The identification of the pathological stage of NSCLC patients is very important for physicians to develop effective treatment plans. The treatment strategy for patients with NSCLC is different according to the stage they are in. Accurate diagnosis determines the effectiveness of treatment strategies and thus avoids misdiagnosis or overtreatment. The efficacy of the treatment is mainly based on whether the values of various diagnostic and decision parameters (e.g., CYFRA21-1, CEA, CA-125, NSE, PSA, etc.) are significantly decreased after the implementation of the treatment strategy. Table 6 show the data of each tumor marker and physiological index recorded in the medical record in sensors, which are the diagnosis and decision parameters of each NSCLC, following the whole process of diagnosis, treatment, and follow-up of typical NSCLC patients in the hospital.

After each phase of treatment, sensors detect and transmit data values for each diagnostic and decision-making parameter of the patient. The physician keeps track of the patient’s recovery based on the system’s recalculation and analysis. From the three highly relevant diagnostic and decision parameters (CYFRA21-1, CEA, CA-125), the values of the corresponding parameters were high at the first examination. The doctor re-diagnosed and adjusted the treatment strategy after each cycle of treatment. After seven treatment cycles, CYFRA21-1 decreased from 4.16 μg/mL to 3.89 μg/mL, CEA decreased from 285.41 ug/L to 21.17 μg/L, and CA-125 decreased from 711.01 KU/L to 178.2 KU/L. This indicates that the patient’s treatment plan was effective after a series of cycles of therapy.

### 4.2. Evaluate Performance

**Figure 10:**
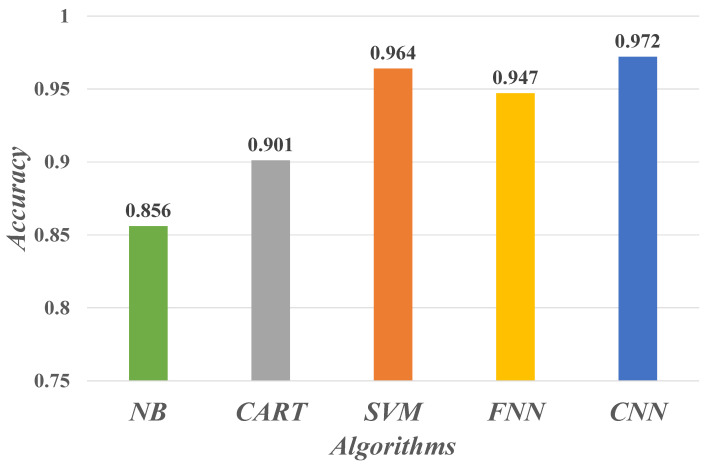
Performance of different machine learning algorithms for tumor staging prediction of NSCLC.

To evaluate the performance of the CNN-based deep learning algorithm for the NSCLC staging prediction model in this paper, we additionally conducted comparative experiments on staging prediction using Naïve Bayes (NB), Classification And Regression Tree (CART), Support Vector Machines (SVM), and Feedforward Neural Network (FNN). Figure 10 show the prediction accuracy of these five learning algorithms. The CNN model shows the best performance in the classification recognition of the sampled data samples, proving the powerful learning and recognition ability of CNN for text semantic features.

The specific parameter configuration of the CNN model is shown in Table 7. In our experiments, we set the number of convolutional kernels to n = 3, and the sizes of convolutional kernels are 3, 4, and 5, respectively. The stochastic gradient descent (SGD) optimization algorithm was used, and the mean value of 10-fold cross-validation was used as the experimental result to ensure the accuracy of the model prediction.

In addition, we also evaluated the performance of the CNN model on the diagnosis of each stage of NSCLC by a set of evaluation metrics, as shown in Table 8. As seen from the AUC value (Area Under ROC Curve), the CNN model used in this paper predicts each NSCLC pathological stage very well. However, due to the small number of case samples in stage I of NSCLC in the whole training set, the samples contain fewer semantic features. Moreover, the model parameters were from the large sample case model of stage III or stage IV, using transfer learning to transfer the knowledge of shared features of each stage of NSCLC, resulting in the low specificity of our model in stage I. Comparatively, the case samples in stage III or stage IV are in the majority, with more knowledge of the features, and the specificity of the model is relatively high. The evaluation metrics sensitivity, specificity, and accuracy are defined in Equations (17)–(19), respectively.

(17) $$Sensitivity=\frac{TP}{TP+FN}$$

(18) $$Specificity=\frac{TN}{TN+FP}$$

(19) $$Accuracy=\frac{TP+TN}{TP+FP+TN+FN}$$

where *TP* is true positive, *FN* is false negative, *TN* is true negative, and *FP* is false positive.

### 4.3. Decision-Making and Discussion

**Figure 11:**
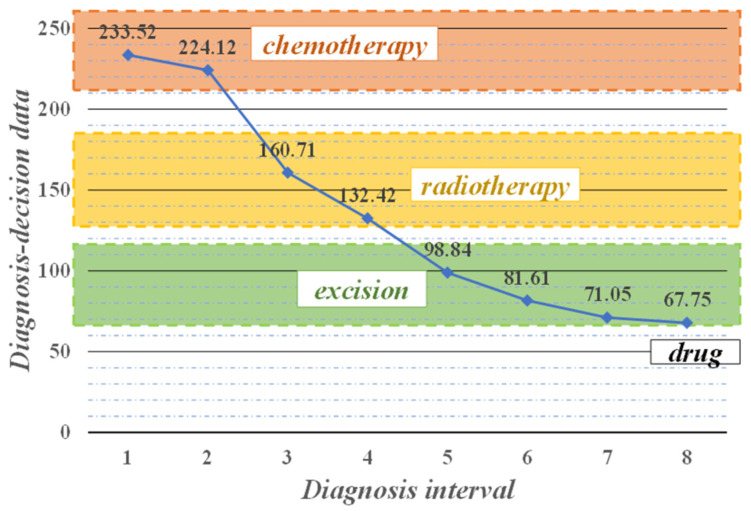
Treatment process of a typical NSCLC patient.

Doctors make dynamic decisions based on the system’s calculations and analysis, adjusting treatment plans and controlling the development of patients’ conditions in real-time, thus achieving precise treatment. Figure 11 show the system of performing the staging diagnosis of a typical NSCLC patient during the treatment process, and recommending the treatment strategy. The physician makes decisions and implements treatment accordingly, from which the patient recovers effectively.

Upon initial diagnosis, the decision value calculated by the system was as high as 233.52 greater than the maximum critical value of 180 in the normal range, indicating that the patient’s NSCLC was in stage IV, which was more serious, and the system recommended chemotherapy strategy. After the physician administered two cycles of chemotherapy to the patient, the system recalculated and analyzed that the decision value decreased and was less than 180, and the patient was in stage III, at which point the recommended treatment strategy was adjusted to radiation therapy because chemotherapy is harmful to the patient’s body and should not be performed for a long time. After two more radiotherapy cycles, the parameter values decreased to between 58 and 119. This indicates that the condition of the patient was in remission and in stage II of NSCLC, which was excised by surgery on removable lesion tissue. The decision value dropped to 67.75 at the eighth diagnosis, at which point the main recommendation was drugs for late treatment. With the help of the assisted diagnosis and decision-making intelligent medical system, doctors can qualitatively analyze the condition of NSCLC patients throughout the whole process. Based on the system’s recommendation of specific and effective treatment strategies after each disease monitoring, the doctor can make accurate decisions and track the patient’s treatment in real-time, thus reducing the patient’s pain during the treatment process and effectively saving the patient’s life.

**Figure 12:**
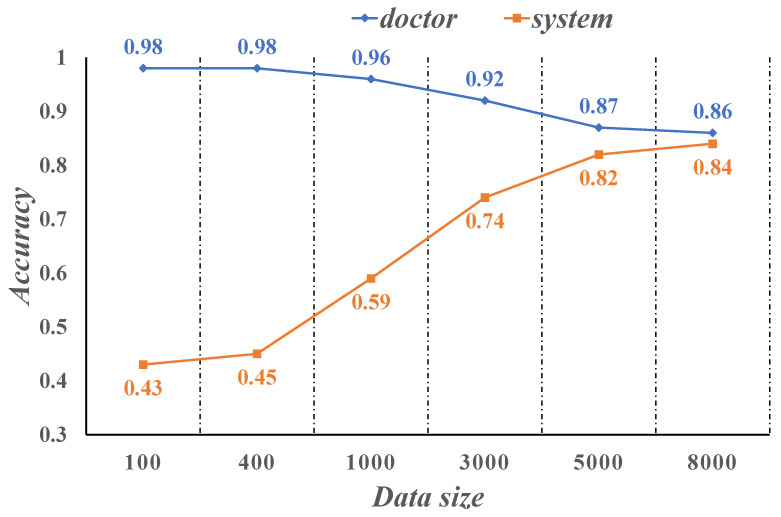
Comparison of doctor and system accuracy.

Figure 12 show the diagnostic accuracy of the physician compared to the intelligent medical system we developed on the sample of NSCLC patient cases. Physician accuracy is as high as 98% when the case sample data size is between 100–500, while the system is only 45% accurate. The accuracy of the system is close to 60% when the case sample data size is 1000. As the case sample data size increases and reaches roughly 5000, the system’s diagnostic correct rate is already as high as 82%, while the doctor’s diagnostic correct rate drops to 87%. When the case sample size reaches 8000, the accuracy of the system is 84%, which is close to the doctor’s diagnostic accuracy of 86%. It can be seen that when the size of the training case sample is large enough, the system can diagnose patients with NSCLC with an accuracy comparable to that of clinicians.

However, trained assisted diagnostic and decision-making intelligent medical systems are only an auxiliary doctor role and can never fully replace clinicians in the diagnosis and decision-making of patients with NSCLC. We expect the auxiliary system to obtain accurate information from the various data recorded in the medical record in sensors to help the physician make the yes or no decision, and improve the efficiency of diagnosis. This relieves doctors from the heavy burden of complicated and trivial diagnostic work in the early period, and the processing of a huge amount of patient case data saves the time and energy of doctors, improves the accuracy of patient diagnosis, and thus avoids misdiagnosis or overtreatment.

## 5. Conclusions

In this paper, we used the Skip-gram model to pre-train word vector matrices on the medical record text data of NSCLC patients, to extract semantic features using the CNN deep learning algorithm, and to use the techniques of transfer learning and dynamic sampling, fusing it into the training process of the system model to build an intelligent medical system for the assisted diagnosis and decision-making of NSCLC. The system accurately stages NSCLC patient case samples to aid in the diagnosis, recommend treatment strategies, and provide physicians with information to support diagnosis and decision-making. After training and testing with data from 2,789,675 patients at three hospitals and comparing experimental analysis with results diagnosed by physicians, the system achieved 84% accuracy, close to that of physician experts, when the case sample data size had reached 8000. The experimental results show that the intelligent medical system we built to assist diagnosis and decision-making can provide doctors with fast and accurate decision-making suggestions, effectively simplify the diagnosis process, saving time, and reducing the load of tedious medical work for doctors.

However, we also recognize that the clinical characteristics of a single clinical data source are limited. In addition, the specificity of our proposed model is relatively low due to the lack of obvious disease characteristics at the first stage of NSCLC, and its performance in this aspect will be further improved in the future. Furthermore, the stability and reliability of the system model requires further research and clinical validation, and other types of NSCLC medical sensors detection information will be adopted by the system for a more comprehensive auxiliary diagnosis of lung cancer. We also hope that the proposed model framework for the assisted diagnosis and decision-making of intelligent medical systems can be applied to other cancers.

## Figures and Tables

**Table 1:** Data collection and type analysis of NSCLC in three hospitals.

Type	Number
Patient information	2,789,675
Outpatient service	968,545
Doctors’ device in outpatient	28,554,590
Hospitalized	1,676,899
Diagnosis	1,124,561
Electronic medical records	5,287,413
Doctors’ device in clinical	31,427,790
Inspection records	179,712
Medical laboratory records	9,483,216
Routine inspection records	24,287,612
Operation records	393,218
Drug records	90,631

**Table 2:** Three highly relevant NSCLC diagnostic and decision parameters and their normal ranges.

Parameter	CYFRA21-1 (μg/mL)	CEA (μg/L)	CA-125 (KU/L)
Range	0–1.80	0–5.00	0–35.00

**Table 3:** Five lowly relevant NSCLC diagnostic and decision parameters and their normal ranges.

Parameter	NSE (μg/mL)	CA242 (KU/L)	PSA (μg/mL)	HGH (μg/mL)	Free-PSA (μg/mL)
Normal data area	0–13.00	0–20.00	0–5.00	0–7.50	0–1.00

**Table 4:** Decision value range for each pathological stage of NSCLC.

Stage Partition	Stage I	Stage II	Stage III	Stage IV
Range	18–57	58–119	119–180	>180

**Table 5:** Mean data of NSCLC diagnosis and decision parameters for each of the three randomly sampled patient groups at five examinations.

CYFRA21-1 (μg/mL)	CEA (μg/L)	CA-125 (KU/L)	NSE (μg/mL)	CA242 (KU/L)	PSA (μg/mL)	HGH (μg/mL)	Free-PSA (μg/mL)	FERRITIN (KU/L)
36.71	3.29	157.64	21	31	0.81	0.51	1.88	154.2
33.58	4.12	189.55	16	24	1.01	0.82	1.45	189.6
40.23	3.15	156.31	27	32	0.95	0.77	1.78	175.8
31.84	3.92	179.32	22	28	1.45	0.48	0.81	193.7
34.53	3.44	198.09	19	31	0.98	0.89	0.57	173.8
1.20	75.48	576.12	33	9	1.22	11.25	21.88	935.7
1.15	82.79	498.32	37	5	1.48	22.82	28.74	854.1
0.91	79.32	524.89	22	6	1.88	19.85	24.32	718.2
1.01	89.11	489.36	24	7	0.99	23.58	26.81	921.5
1.03	84.12	518.88	27	4	1.57	18.78	37.58	814.6
1.22	6.77	116.32	31	21	7.22	6.51	0.12	258.9
1.41	8.24	97.54	36	26	7.52	5.32	0.55	322.7
1.32	16.78	104.58	35	29	7.14	4.87	0.17	278.9
1.20	22.12	99.28	28	24	8.56	5.99	0.45	341.8
1.19	17.95	89.65	21	22	8.47	6.02	0.67	304.8

**Table 6:** The data of various diagnostic and decision parameters during the treatment of a typical NSCLC patient.

	CYFRA21-1 (μg/mL)	CEA (μg/L)	CA-125 (KU/L)	NSE (μg/mL)	CA242 (KU/L)	PSA (μg/mL)	HGH (μg/mL)	Free-PSA (μg/mL)	FERRITIN (KU/L)
1	4.16	285.41	711.01	34	8	1.12	10.25	58.81	835.7
2	5.57	277.99	688.81	36	5	1.42	11.21	49.71	754.1
3	3.55	257.15	521.42	27	6	1.86	12.15	48.22	738.2
4	4.28	231.44	461.56	25	6	1.29	11.20	47.55	622.1
5	3.47	184.88	408.18	36	5	1.54	15.71	38.51	422.6
6	4.84	128.11	321.88	27	7	1.68	13.88	35.12	351.8
7	5.17	62.89	295.10	38	6	1.71	12.51	11.6	211.1
8	3.89	21.17	178.20	21	5	1.55	13.61	7.1	209.7

**Table 7:** The CNN model parameters configuration.

Parameters	Description
layer 1	word vector matrix
layer 2	convolutional layer with multi-scale kernels, 3 kernels, kernel size [3,4,5]
layer 3	1-max pooling
layer 4	full connection with dropout = 0.5 and softmax output
epoch size	256
optimizer	stochastic gradient descent (SGD)
k-fold	10

**Table 8:** The performance of CNN models in four NSCLC stages.

Category	Sensitivity (%)	Specificity (%)	Accuracy (%)	AUC
Stage I	92.04	91.34	92.20	0.93
Stage II	93.20	92.30	94.17	0.94
Stage III	94.60	95.85	97.50	0.97
Stage IV	96.83	96.79	95.94	0.96
